# Long non-coding RNA LUADT1 promotes nasopharyngeal carcinoma cell proliferation and invasion by downregulating miR-1207-5p

**DOI:** 10.1080/21655979.2021.2001952

**Published:** 2021-12-01

**Authors:** Ning Jiang, Lijun Zhao, Dan Zong, Li Yin, Lirong Wu, Cheng Chen, Xue Song, Qian Zhang, Xuesong Jiang, Xia He, Jifeng Feng

**Affiliations:** a Department of Radiation Oncology, The Affiliated Cancer Hospital of Nanjing Medical University & Jiangsu Cancer Hospital & Jiangsu Institute of Cancer Research, Nanjing, China; b Department of Medical Oncology, The Affiliated Cancer Hospital of Nanjing Medical University & Jiangsu Cancer Hospital & Jiangsu Institute of Cancer Research, Nanjing, China

**Keywords:** Nasopharyngeal carcinoma, lncRNA-LUADT1, miR-1207-5p, TEAD1, Hippo/YAP signaling pathway

## Abstract

Nasopharyngeal carcinoma (NPC) is a typical type of malignant tumor. This research paper aims to study the function and mechanism of long non-coding RNA lung adenocarcinoma-related transcript 1 (lncRNA-LUADT1) in the progression of NPC. In this study, the expressions of lncRNA-LUADT1, miR-1207-5p, and TEAD1 in NPC tissues and cell lines were detected by RT-qPCR. Initially, the expression of lncRNA-LUADT1 and TEAD1 were significantly up-regulated in NPC tissues and cells, while miR-1207-5p was significantly down-regulated. Next, miR-1207-5p was confirmed to bind to lncRNA-LUADT1 or TEAD1 by bioinformatics and luciferase reporter assay. In addition, after interfering with lncRNA-LUADT1 expression, experiments of CCK8, EDU staining, and Transwell invasion were used to detect proliferation, invasion, and migration of NPC cells. The results showed that interfering with lncRNA-LUADT1 expression could inhibit the proliferation, invasion, and migration of NPC cells. Western blot showed that lncRNA-LUADT1 knockdown significantly decreased the expression of Hippo/YAP pathway protein (YAP1 and TAZ). However, interfering with the expression of miR-1207-5p reversed these results. In addition, the nude mouse tumor formation experiment suggested that low-expressed lncRNA-LUADT1 reduced the volume and weight of tumor tissues. In summary, lncRNA-LUADT1 down-regulation could inhibit NPC cell proliferation and invasion, which may be achieved through regulating miR-1207-5p expression and affecting TEAD1 expression, thus inhibiting the activation of Hippo/YAP signaling pathway.

## 1. Introduction

Nasopharyngeal carcinoma (NPC) is a malignant tumor that occurs in the head and neck and has a high incidence in China. NPC incidences are exceptionally high in southern China, mainly in provinces such as Guangdong, Guangxi, and Hunan, accounting for about 80% of the global cases [1,2]. Current studies have demonstrated various factors related to NPC onset, including Epstein–Barr Virus (EBV) infection, genetics, diet, and environment [3]. Poorly differentiated squamous cell carcinoma classified domestically and undifferentiated non-keratinizing carcinoma classified by WHO are the most common pathological types of NPC [4]. Radiotherapy is still the preferred treatment method for NPC at present. With the development of stereotactic radiotherapy technology, NPC patients’ local control rate and survival time have been generally improved, and the five-year survival rate has been maintained at about 70% [5]. However, NPC metastasizes early and can easily relapse, so patients with advanced NPC have a survival rate of only 30%–40% [6]. Local reappearance and remote metastases are the leading causes of treatment failure and the main challenges. However, which key components and molecular mechanisms participate in proliferation, metastasis, and invasion of NPC remains unclear. Therefore, a comprehensive analysis of the molecular mechanisms in NPC recurrence and metastasis is significant for advanced NPC patients that may improve their survival rates.

Long non-coding RNAs (lncRNAs) are classed in the endogenous RNA division with a length greater than 200 nucleotides that have entirely lost or partly losing the encoding ability to small peptides [7–9]. A variety of abnormally expressed LncRNAs have been identified in NPC [10]. Wang et al. [11] found that lncRNA PVT1 was upregulated in NPC and knockdown of lncRNA PVT1 reduced NPC cell proliferation and tumorigenesis. Liu et al. [12] showed that LncRNA SNHG5 promoted NPC progression by regulating the miR-1179/HMGB3 axis. Xu et al. [13] reported that lncRNA SNHG7 could promote NPC through epithelial–mesenchymal transition. The latest research reports that transcript 1 (LUADT1)-associated lung adenocarcinoma is a carcinogenic lncRNA, which promotes lung adenocarcinoma cell proliferation by inhibiting p27 epigenetics [14]. Up-regulated lncRNA-LUADT1 expression is seen in colon cancer. It is intimately linked to tumor size, lymph node metastasis, and TNM classification, and the high-expressed lncRNA-LUADT1 indicates that the overall survival rate is low [15].

Meanwhile, lncRNA can exert its function by combining microRNA (miRNA). MicroRNA (miRNA) are non-coding short RNA pieces that bind to targeted mRNA 3ʹ-UTR to promote or inhibit cellular processes [16]. Gao et al. showed that promoting oral squamous cell carcinoma cell proliferation could be completed by lncRNA-LUADT1 by miR-34a/GAS1 axis regulation [17]. Wang et al. discovered that small cell lung cancer Twist1 up-regulation could be done by lncRNA-LUADT1 inducing miR-15a-3p, thereby promoting its occurrence and development [18]. Bioinformatics predicts that lncRNA-LUADT1 can bind to miR-1270-5p, which has been reported to be regulated by LncRNA319, thereby encouraging the development of NPC [19]. Though no research has stated lncRNA-LUADT1 and miR-1270-5p targeting effects, lncRNA-LUADT1/miR-1270-5p effects on the pathological process of NPC and its mechanism need to be further studied. This article discussed the relationship between lncRNA-LUADT1 and miR-1270-5p in NPC, its mechanism, and impact on NPC proliferation, migration, and invasion, providing an up-to-date theoretical basis for NPC treatment.

## 2. Materials and methods

### 2.1. Clinical specimen collection

We collected 79 cases of tumor tissue (NPC) and 10 cases of normal tissues adjacent to the tumor (Normal) from patients with NPC in The Affiliated Cancer Hospital of Nanjing Medical University from February 2017 to August 2019. The average age of patients is 55.86 ± 14.58 years. All specimens were pathologically diagnosed and kept in liquid nitrogen. Approval was given to this study by the ethics committee of our hospital with signed informed consent forms from all involved patients.

### 2.2. Cell culture

Human normal nasopharyngeal epithelial cell line NP69, NPC cell lines HONE-1, HNE-1, CNE1, CNE2 were acquired from the Shanghai Institute of Cell Biology, Chinese Academy of Sciences cell bank. NP69 and CNE1 were cultured by using RPMI-1640 medium (GIBCO, USA), while CNE2, HONE-1, and HNE-1 cells were cultured utilizing DMEM medium (GIBCO, USA). Both medium contained 10% fetal bovine serum (FBS; Hyclone, USA), 100 U/ml penicillin, and 100 μg/ml streptomycin. The cells were then placed in a 5% CO_2_ incubator at 37°C.

### 2.3. Cell transfection

Twelve-well plates were used to inoculate logarithmic growth phase cells till the cell confluence reached 50–60%. Cells were then transfected with si-NC, si-lncRNA-LUADT1 (si-LUADT1), NC mimics, miR-1207-5p mimics, NC inhibitor, and miR-1207-5p inhibitor by Lipofectamine-2000 reagent (Thermo, USA). The culture medium was substituted with a fresh medium following 6 h of transfection before continuing to culture and incubated for 48 h. si-NC, si-LUADT1, NC mimics, miR-1207-5p mimics, NC inhibitor, and miR-1207-5p inhibitor were acquired from RIBOBIO (Guangzhou, China).

### 2.4. CCK-8 assay

Cells were inoculated in a plate with 96-well of 5000 cells/well, with three replicates in each well. After inoculation and adherence, transfection was done for the cells of each group for 24, 48, and 72 h before adding 10 μL of CCK-8 reagent (Beyotime, China) to respective wells and incubation in a 5% CO_2_, 37°C incubators for 2 h. A microplate reader was used to detect each well’s optical density (OD) at the 450 nm wavelength. Testing was done in triplicate.

### 2.5. EDU detection

DNA synthesis of each group of cells was evaluated by the EDU kit (Tiangen, China) using EDU immunofluorescence analysis. Following 48 h of cell transfection, inoculation of the cells was done in a 96-well plate of 1 × 10^4^ cells/well. After the cell adherence, they were replaced in a 50 μmol/L EDU medium and incubated for 4 h before fixation with 4% paraformaldehyde for 15 min and 10 min 0.2% glycine incubation. Later, PBS was used to rinse the cells twice, permeabilized by 0.5% Triton X-100, rinsed again with PBS before incubation in Apollo staining reaction solution for 30 min in the dark. Hereafter, cell incubation was done with Hoechst in the dark for 10 min following PBS rinsing and then rinsed with 0.5% Triton X-100 3 times, and finally, they were imaged under a fluorescence microscope.

### 2.6. Transwell migration assays

After transfection, we collected the cells and inoculated 200 μL into the Transwell chamber upper layer, with a complete medium filling the chamber lower layer. After 24 h of culture, 4% paraformaldehyde was used for a 30-min fixation with 0.1% crystal violet used to stain for 10 min. A random selection of 10 fields was made for picturing the lower surface of the polycarbonate membrane before cell count, and statistical analysis was performed.

### 2.7. Transwell invasion assays

After hydrating the matrix gel (BD Biosciences, USA) in a serum-free medium, it was diluted and spread evenly in the upper Transwell chamber (Fermentas, USA), with three replicates in each group. We added 100 μl of 1 × 10^6^ cells/ml cell suspension to the upper section and a complete medium to the lower section before incubation in 5% CO_2_ for 24 h at 37°C. The upper chamber was taken out, and methanol was used to fix for 30 min before staining with 0.1% crystal violet for 30 min. The microscope was used to count the quantity of cells that passed through the filter (the average number from eight fields was taken as the average value).

### 2.8. Dual luciferase reporter assay

The online target gene prediction website ENCORI (http://starbase.sysu.edu.cn/) showed binding sites of lncRNA-LUADT1 and miR-1207-5p. The target gene prediction online website TargetScan (http://www.targetscan. org/vert_72/) revealed the existence of miR-1207-5p binding sites with TEAD1 as well. The luciferase expression vectors WT-LUADT1 and MUT-LUADT1 for the wild-type and mutant gene targets lncRNA-LUADT1 were constructed, respectively. The luciferase expression vectors WT-TEAD1 and MUT-TEAD1 for the target gene TEAD1 were co-transfected with NC mimic, and miR-1207-5p mimics into 293 T cells, respectively, LipofectamineTM 2000 (Yeasen, China) to count their luciferase activity. Experimentation was done in triplicate.

### 2.9. qRT-PCR

Extraction of total RNA was completed utilizing the Trizol reagent (Invitrogen). The cDNA Transcription Kit (ABI) was used to reversely transcribe RNA to cDNA. The rapid quantitative PCR was achieved on the 7500 Real-Time PCR System (Applied Biosystem, USA) utilizing SYBRH Select Master Mix (Invitrogen). The parameters were as follows: 95°C 5 min, then 95°C 15 s, 55°C 30 s, and 60°C 1 min, a total of 40 cycles. The GAPDH or U6 expression standardized all results with the quantitative analysis selecting the 2^−ΔΔCt^ method. Primer sequences are included in Table 1.

**Table 1.** Primer sequences

Primer	Sequences
lncRNA-LUADT1	F 5ʹ- TTTCCGTTCAGCAAATCCACAC-3’
	R 5ʹ- TTAGGTCCAGCACTGTTATCCA-3’
GAPDH	F 5ʹ-AACGGATTTGGTCGTATTG-3’
	R 5ʹ-GGAAGATGGTGATGGGATT-3’
miR-1207-5p	F 5ʹ-CGCCGATATTGGTCGGATGAT-3’
	R 5ʹ-TTCAGCGTACACCTAATCGGTATG-3’
U6	F 5ʹ-CCGTCGTAGGCAGCCTCTACGCT-3’
	R 5ʹ-GCATCCAACGTACGCTCATCGT-3’

### 2.10. Western blot

RIPA lysis buffer, including 1% phenylmethylsulfonyl fluoride (PMSF), was added to each group of cells to extract protein, measured by the BCA kit (Thermo, USA). Total protein was heated to 100°C and incubated for 5 min before being separated by SDS-polyacrylamide gel electrophoresis (120 v, 100 min) and finally transferred to a PVDF membrane (300 mA, 80 min). After blocking with 5% BSA to, the membrane was incubated with primary rabbit monoclonal antibody TEAD1 (1:1000, ab109080), rabbit monoclonal antibody YAP1 (1:1000, ab76252), rabbit monoclonal antibody LATS1 (1:1000, ab243656), rabbit polyclonal antibody TAZ (1:500, ab224239), and rabbit monoclonal antibody GAPDH (1:1000, ab181602) at 4°C overnight. The membrane was rinsed 3 times the following day before incubation with a secondary antibody with horseradish peroxidase (HRP) conjugated polyclonal goat anti-rabbit IgG (1:10,000, ab6721). The membrane was then set at room temperature for 1 h and cleaned various times. After applying the luminescent liquid, the membrane was put into a chemiluminescence instrument for imaging, which was then analyzed its gray value by Image J.

### 2.11. Xenograft tumor in nude mice

Six female BALB/c nude mice (age 4–5 weeks, 18–20 g) were randomly divided into 2 groups (3 in each group). The HNE-1 cells transfected with si-LUADT1 or si-NC in the exponential growth phase were collected. Suspensions were made at a density of 1 × 10^7^ cells/mL, and 100 μL were inoculated in the axilla of nude mice (1 × 10^6^ cells/mouse). Once the tumor nodules were palpable, the long diameter (a) and short diameter (b) of the tumor were measured and recorded every 3 days. Calculate the volume: v = (a × b^2^)/2, and plot the growth curve. All mice were executed 30 days after inoculation, and the tumors were excised and weighed.

### 2.12. Statistical analysis

SPSS 22.0 software was employed for statistically obtained data analysis. Mean ± standard deviation (SD) was applied to express the measurement data. Variations among both groups were evaluated using the *t* test. Comparisons among multiple groups were performed by one-way analysis (ANOVA), followed by Tukey’s post-hoc test. Experimentation was completed no less than 3 times. A value of P < 0.05 represents statistical significance.

## 3. Results

### 3.1. lncRNA-LUADT1 is substantially upregulated in nasopharyngeal carcinoma tissues and cells

**Figure 1.**
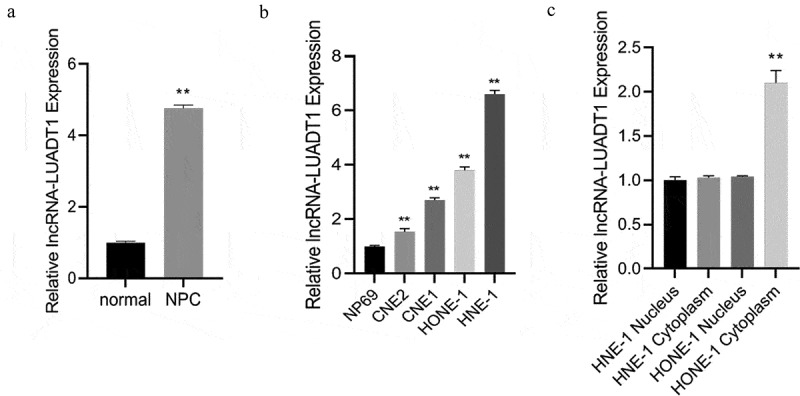
lncRNA-LUADT1 expression levels in tissues and cells of nasopharyngeal carcinoma patients

First, expression levels of lncRNA-LUADT1 in NPC tissues and cell lines were tested. The findings demonstrated a notably higher lncRNA-LUADT1 expression in NPC tissues than adjacent tissues (Figure 1(a)). In addition, lncRNA-LUADT1 was expressed higher in NPC cell lines (HONE-1, HNE-1, CNE1, CNE2) than normal nasopharyngeal epithelial cells (NP69) (Figure 1(b)). Outcomes have confirmed that lncRNA-LUADT1 was expressed during the occurrence of NPC, and it was further detected that in HNE-1 and HONE-1 cells, lncRNA-LUADT1 was higher-expressed in the cytoplasm of HONE-1 cells (Figure 1(c)).

A, qRT-PCR for evaluating the lncRNA-LUADT1 expression in nasopharyngeal carcinoma cancer tissues and adjacent tissues; **P < 0.01 vs. normal group; B, qRT-PCR for measuring lncRNA-LUADT1 expression in nasopharyngeal carcinoma cell lines (HONE-1, HNE) −1, CNE1, CNE2) and normal nasopharyngeal epithelial cells (NP69); **P < 0.01 vs. NP69 group; C, lncRNA-LUADT1 expressed in the nucleus and cytoplasm of HNE-1 and HONE-1 cells; **P < 0.01 vs. HONE-1 nucleus group.

### 3.2. Knocking down LUADT1 expression can inhibit the nasopharyngeal carcinoma process

**Figure 2.**
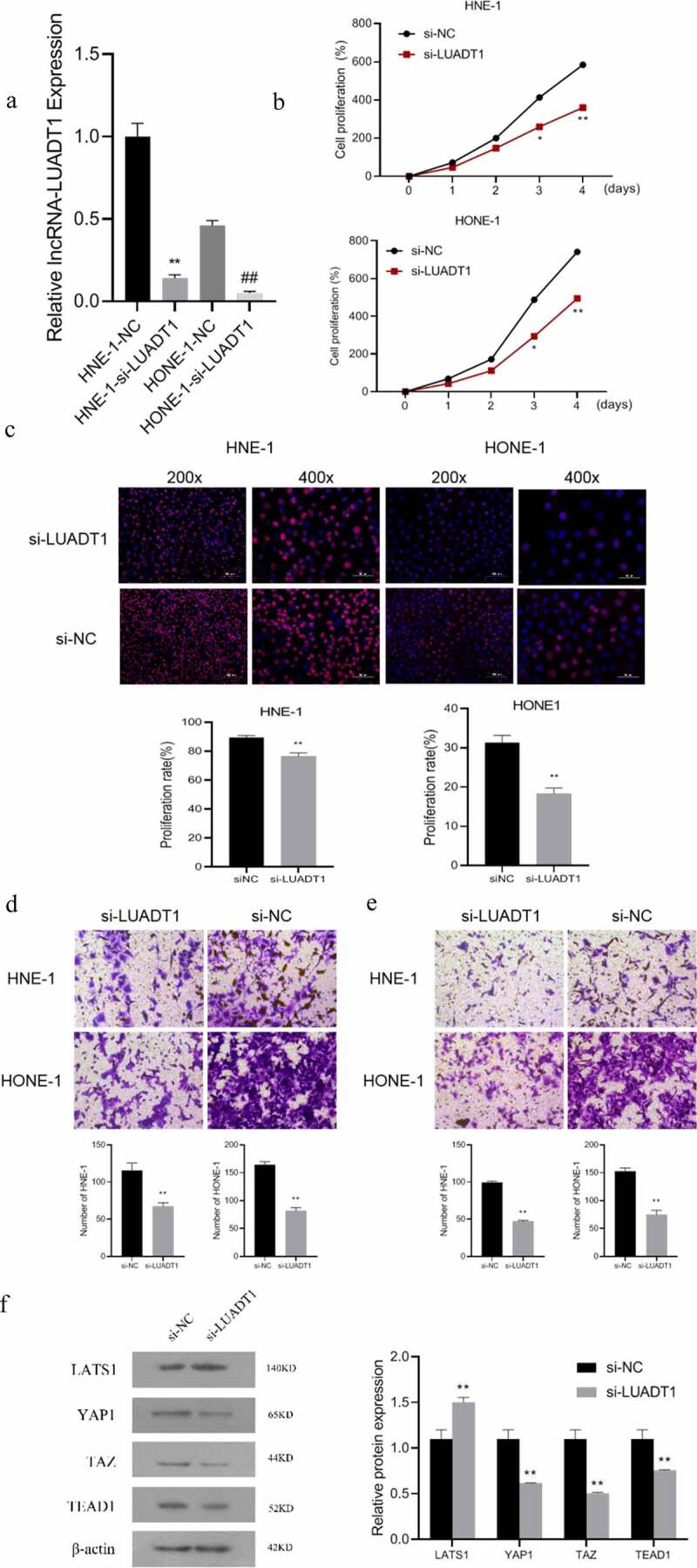
The effect of LUADT1 expression on the nasopharyngeal carcinoma process

For exploring the biological functions of lncRNA-LUADT1, we performed the loss-of-function experiment by interfering with the expression of lncRNA-LUADT1. After transfecting si-NC and si-LUADT1 in HNE-1 cells and HONE-1 cells, the expression of lncRNA-LUADT1 in HNE-1 cells and HONE-1 cells decreased markedly (P < 0.05) (Figure 2(a)), indicating the successful transfection. CCK-8, EdU with Transwell further applied to detect cell viability, cell proliferation, and cell invasion, respectively. It proved that after knocking down lncRNA-LUADT1, cell viability, proliferation, etc., invasion and migration of HNE-1 and HONE-1 cells were suppressed (Figure 2(b-d)).

After determining the function of lncRNA-LUADT1 in NPC, we further explored the molecular mechanism of its involvement. It has been shown that the Hippo/YAP signaling pathway is involved in the development of NPC [20]. Subsequently, Western blot detection verifies the expression level of LATS1 in the si-LUADT1 group was distinctly higher, while the expression of YAP1, TAZ, and TEAD1 were significantly lower compared with the si-NC group (Figure 2(f)), displaying that knocking down lncRNA-LUADT1 can inhibit Hippo/YAP signaling pathway activation.

A, The expression of lncRNA-LUADT1 after transfecting HNE-1 and HONE-1 cells with si-NC and si-LUADT1, **P < 0.01 vs. HNE-1-NC group, ^##^P < 0.01 vs. HONE-1 -NC group; B, CCK-8 methods for assessing the cell viability in HNE-1 and HONE-1 cells; C, EDU for observing the cell proliferation in HNE-1 and HONE-1 cells; D, The cell migration ability in HNE-1 and HONE-1 groups via Transwell experiment; E, HNE-1, and HONE-1 group cell invasion ability by Transwell experiment; F, Western blot for evaluating expressed proteins related to Hippo/YAP signaling pathway; **P < 0.01 vs. si-NC group

### 3.3. lncRNA-LUADT1 targets miR-1207-5p in nasopharyngeal carcinoma

**Figure 3.**
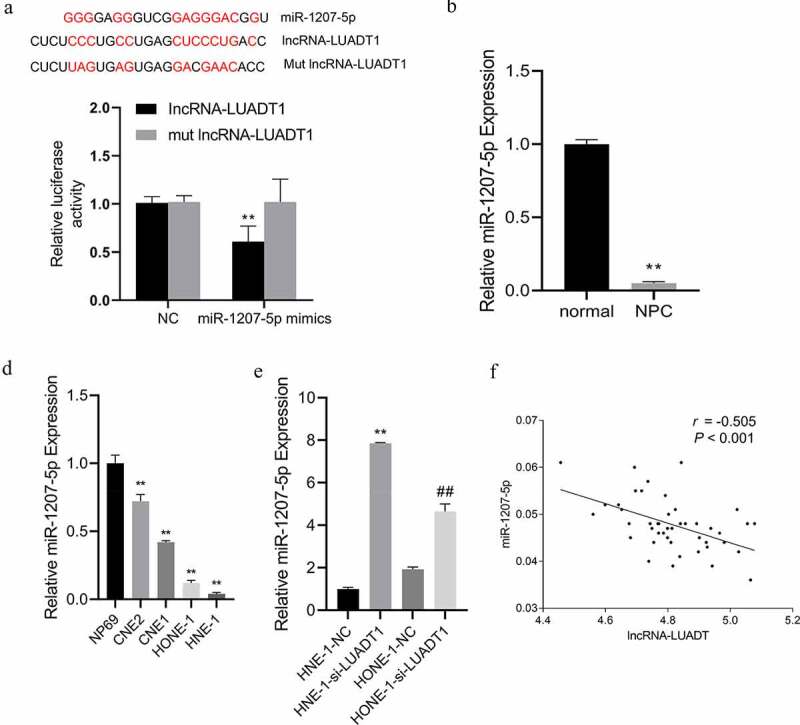
The targeting effect of lncRNA-LUADT1 on miR-1207-5p

To further study the mechanism of how lncRNA-LUADT1 affects NPC cells, we first used the target gene prediction online website ENCORI (http://starbase.sysu.edu.cn/) and found binding sites miR-1207-5p and lncRNA-LUADT1. And it was further confirmed by the luciferase reporter gene experiment that miR-1207-5p mimics and miR-1207-5p could notably suppress luciferase activity of the lncRNA-LUADT1 wild-type plasmid but not the MUT-lncRNA-LUADT1 vector (Figure 3(a)). The distinctly low-expressed miR-1207-5p was also found in NPC tissues and cell lines (Figure 3(b-c)). After inhibiting the expression of lncRNA-LUADT1 in HNE-1 and HONE-1 cells, expressed miR-1207-5p was up-regulated (Figure 3(d)). Further correlation analysis revealed that lncRNA-LUADT1 and miR-1207-5p expression was distinctly negatively correlated (Figure 3(e)), suggesting that lncRNA-LUADT1 can bind miR-1207-5p competitively, thereby negatively regulating its expression.

A, Binding sites of lncRNA-LUADT1 and miR-1207-5p using dual-luciferase reporter gene; B, qRT-PCR for measuring miR-1207-5p expression in nasopharyngeal carcinoma patient tissues and adjacent tissues, **P < 0.01 vs. normal group; C, qRT-PCR for evaluating the miR-1207-5p expression in different nasopharyngeal carcinoma cells and normal nasopharyngeal epithelial cells, **P < 0.01 vs. NP69 group; D, miR-1207-5p expression after transfection of NHE-1 and HONE-1 cells with si-NC and si-LUADT1, **P < 0.01 vs. HNE-1-NC group, ^##^P < 0.01 vs. HONE-1-NC group; E, Correlation analysis of the lncRNA-LUADT1 and miR-1207-5p.

### 3.4. TEAD1 is the target gene of miR-1207-5p

**Figure 4.**
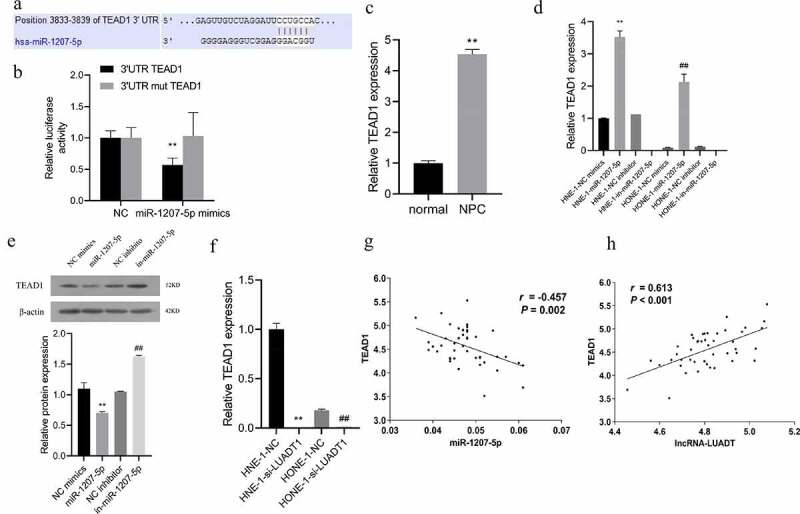
TEAD1 is the target gene of miR-1207-5p

Deeply, it was predicted that TEAD1 was a target gene of miR-1207-5p through the TargetScan (http://www.targetscan.org/vert_72/) (Figure 4(a)). The dual-luciferase reporter assay also reviewed markedly suppressed luciferase activity after miR-1207-5p mimics was co-transfected with the wild-type plasmid containing the TEAD1 3ʹ-UTR (Figure 4(b)). And the TEAD1 expression in NPC tissues is notably lower than in neighboring tissues (Figure 4(c)). Subsequently, for further verification of the targeting relationship among miR-1207-5p and TEAD1, we interfered with or overexpressed miR-1207-5p in HNE-1 and HONE-1 cells and discovered a markedly decreased TEAD1 expression after overexpressing miR-1207-5p. However, its expression was raised distinctly after miR-1207-5p inhibition (Figure 4(d-e)). After interfering with LUADT1, the expression of TEAD1 decreased significantly (Figure 4(f)). Correlation analysis discovered a negative correlation between miR-1207-5p and TEAD1 expression (Figure 4(g)), while LUADT1 and TEAD1 expression was evidently positively correlated (Figure 4(h)). All these results displayed that lncRNA-LUADT1 can competitively bind to miR-1207-5p and regulate TEAD1 expression.

A, The binding sites of miR-1207-5p and TEAD1predicted by TargetScan website; B, Verification of the binding of miR-1207-5p and TEAD1 by dual-luciferase reporter gene; C, qRT-PCR for determining TEAD1 expression in tissues of patients with nasopharyngeal carcinoma, **P < 0.01 vs. normal group; D, qRT-PCR for evaluating TEAD1 expression in each group of nasopharyngeal carcinoma cells, **P < 0.01 vs. HNE-1-NC mimics group, ^##^P < 0.01 vs. HONE-1-NC mimics group; E, Assessment of TEAD1 expression in each group of cells with Western blot; F, qRT-PCR for measuring TEAD1 expression in NHE-1 and HONE-1 cells, **P < 0.01 vs. HNE-1-NC group, ^##^P < 0.01 vs. HONE-1-NC group; G, Correlation analysis of the relationship between miR-1207-5p and TEAD1; H, Correlation analysis of the relationship between lncRNA-LUADT1 and TEAD1.

### 3.5. lncRNA-LUADT1 regulates the miR-1207-5p expression and affects the nasopharyngeal carcinoma process

**Figure 5.**
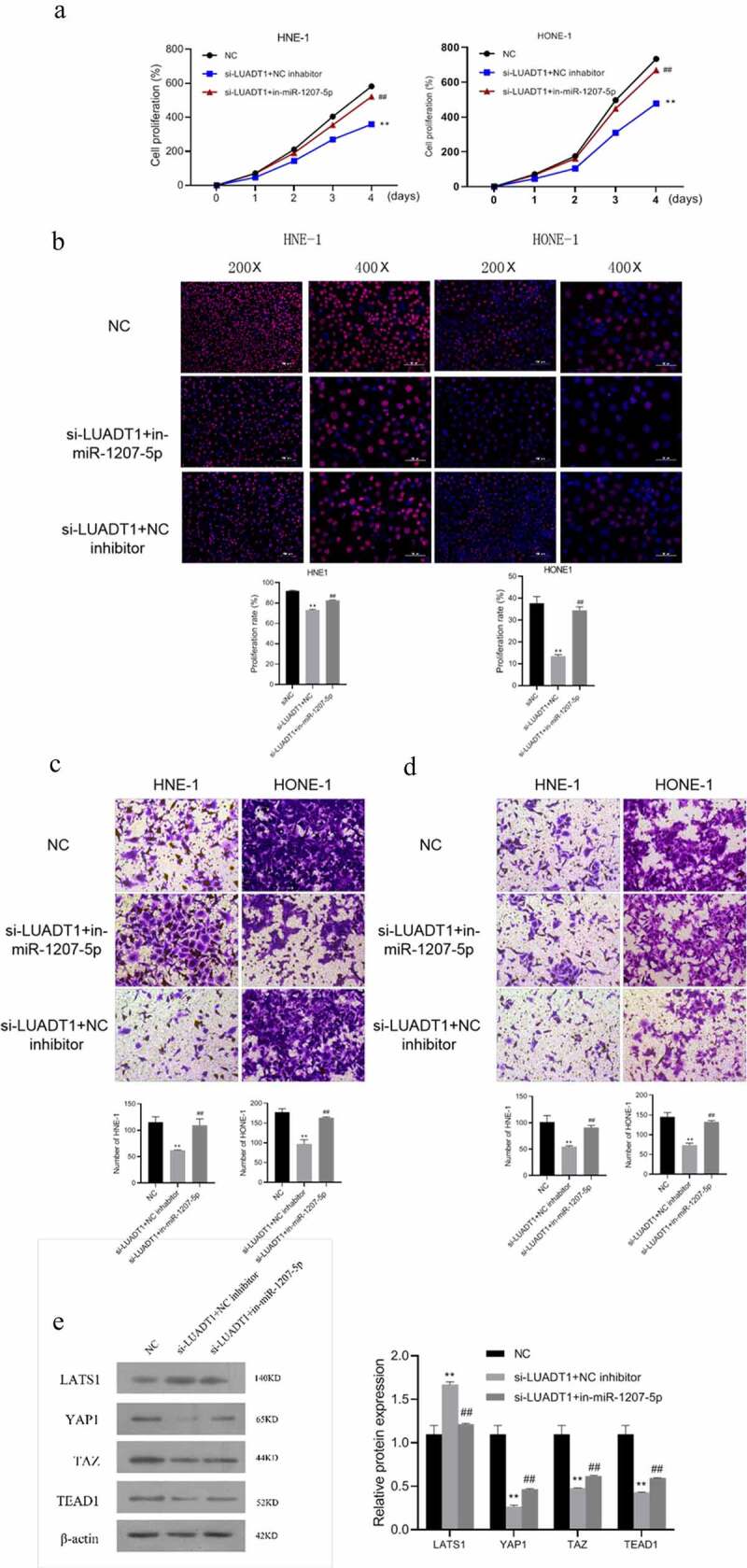
The effect of lncRNA-LUADT1 and miR-1207-5p on the nasopharyngeal carcinoma process

We conducted a cell recovery experiment to investigate lncRNA-LUADT1/miR-1207-5p function in the NPC process by transfecting si-LUADT1 or miR-1207-5p inhibitor into HNE-1 and HONE-1 cells. The findings revealed down-regulated miR-1207-5p eliminated the inhibitory effects of si-LUADT1 on the viability, proliferation, migration, and invasion of NHE-1 and HONE-1 cells (Figure 5(a-d)). Meanwhile, the si-LUADT1+ in-miR-1207-5p group had a distinctly lower LATS1 expression level than the si-LUADT1 group. Still, higher protein expression levels of YAP1, TAZ, and TEAD1 (Figure 5(e)) demonstrate that lncRNA-LUADT1 can affect the proliferation, migration, and invasion of NPC cells and the Hippo/YAP pathway by regulating miR-1207-5p.

A, Detection HNE-1 and HONE-1 cell viability using CCK-8; B, Assessment of the proliferation ability of HNE-1 and HONE-1 cells by EDU; C, Transwell test for verifying the migration ability of HNE-1 and HONE-1 cells; D, Transwell Experiment for evaluating the invasion ability of HNE-1 and HONE-1 cells; E, Western blot for determining the related protein expression of Hippo/YAP signaling pathway in HNE-1 cells; **P < 0.01 vs. si-NC group; ^##^P < 0.01 vs. si-LUADT1+ NC inhibitor

### 3.6. Knocking down lncRNA-LUADT1 can inhibit tumor growth in nude mice

**Figure 6.**
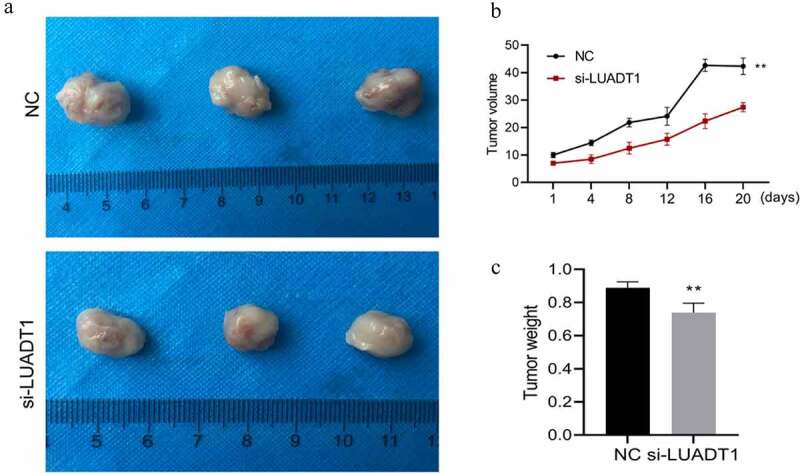
lncRNA-LUADT1 effect on tumor growth in nude mice

Finally, we verified the effect of lncRNA-LUADT1 on NPC tumors in vivo. We subcutaneously injected si-NC and si-LUADT1 transfected HNE-1 cells into the left axilla of nude mice to build an in vivo nude mouse tumorigenesis model. Tumor volume and tumor weight in the si-LUADT1 group were exhibited markedly lower than the si-NC group (p < 0.05) (Figure 6(a-c)), suggesting that knocking down LUADT1 can inhibit tumor growth in nude mice.

- Tumor size after interfering with lncRNA-LUADT1; B. Tumor volume after interfering with lncRNA-LUADT1; C. Tumor weight after interfering with lncRNA-LUADT1. **P < 0.01 vs. NC group

## 4. Discussion

NPC is a conventional malignant tumor on the head and neck in China, usually highly malignant. People aged 40–50 years are the high incidence group of NPC, and it increases with age and has a worse prognosis [1]. NPC has an insidious onset, so it is not easy to be found. Meanwhile, due to the fast proliferate, invade, and metastasize, as well as the particular anatomical location of NPC, along with concentrated and abundant surrounding blood vessels and lymphatic vessels, about 75% of patients had reached the late stage when they were found to have lymph nodes [21,22]. The pathogenesis of NPC is complex, mainly caused by multiple interactions, multiple mechanisms, multiple pathways, multiple gene expression abnormalities, and regulatory imbalances. With more studies about lncRNA recently, some lncRNAs reveal to exert a vital function in disease occurrence and development, with different roles in different parts and different diseases. A study has reported that up-regulated lncRNA-LUADT1 is seen in small cell lung cancer that up-regulates Twist1 expression by sponging miR-15a-3p, thus encouraging cancer cell invasion and migration [23]. This study showed evidence of highly expressed lncRNA-LUADT1 in NPC tissues, and cell lines, representing that lncRNA-LUADT1 may be involved in the occurrence of NPC.

Furthermore, we found that suppressing lncRNA-LUADT1 expression can evidently inhibit NPC cell proliferation, migration, and invasion. In vivo, nude mice experiment also proved lncRNA-LUADT1 expression interference can notably impede the growth of NPC tumors. These results displayed that lncRNA-LUADT1 can prohibit NPC occurrence and development.

Current studies reveal that lncRNA can positively or negatively regulate the expression of coding genes by regulating cell proliferation and differentiation, inducing chromatin remodeling, and adsorbing miRNAs [24]. One important regulatory mechanism of lncRNA is that being a competitive endogenous Sex RNA (ceRNA) interacts with miRNA, thereby regulating target gene expression [25]. For example, lncRNA-XIST could be used as an endogenous ‘sponge’ to directly bind miR-92b to promote hepatocellular carcinoma progress [26]. LINC02570 adsorbs miR-4649-3p to up-regulate SREBP1 to promote NPC progression [27]. To clarify whether lncRNA-LUADT1 regulates like this, we searched the target gene online prediction database for obvious binding site prediction among miR-1207-5p and lncRNA-LUADT1. We performed dual-luciferase reporter assay to verify that lncRNA-LUADT1 can target and regulate miR-1207-5p expression. Simultaneously, it was found that low miR-1207-5p expressions eliminates low-expressed lncRNA-LUADT1 inhibitory effects on NPC cell proliferation, migration, and invasion.

MiRNA mainly combines with specific site of 3ʹUTR of target gene and thus regulates gene expression and affect the biological cell activity of the cell. Therefore, we used the online prediction database TargetScan to predict the target gene of miR-1207-5p, and TEAD1 was the potential target gene. The prediction results were also verified by the dual-luciferase reporter assay, which demonstrated that TEAD1 is a direct target gene of miR-1207-5p. Research has reported that TEAD1 is up-regulated in gastric cancer and regulated negatively by miR-4269. Its elevated expression may possibly promote gastric cancer cell proliferation [28], that is equivalent for liver cancer cells [29]. We also found that NPC tissues have a high expression of TEAD1 with overexpression of miR-1207-5p in NPC cells, notably inhibiting TEAD1 expression, which has the same effect of interfering with the expression of lncRNA-LUADT1. In addition, TEAD1 negatively correlates with miR-1207-5p expression in NPC tissues and positively correlates with the lncRNA-LUADT1 expression. Findings indicated lncRNA-LUADT1 competitive binding of miR-1207-5p in NPC cells to regulate TEAD1 expression.

The Hippo/YAP signaling pathway was discovered from the study about the genetic function of Drosophila. As a cell growth inhibitory pathway, it regulates the size of tissues and organs by maintaining the balance of cell proliferation and apoptosis [30], involving cell proliferation, survival, cell fate determination, and regeneration [31]. It is a three-level cascade with a mammalian sterile 20-like kinase 1/2 (MST1/2), large tumor suppressor 1/2 (LATS1/2), and yes-associated protein (YAP) like composition [32]. The critical elements of the Hippo pathway include MST, SAV, LATS, and MOB. Yes-associated protein (YAP) portray a transcriptional co-activator of the Hippo/YAP pathway. When the Hippo pathway is dysfunctional, unphosphorylated YAP enters the nucleus and binds to transcription factors such as TEADs and SMAD to activate target gene expression and promote cell proliferation, and ultimately participate in tumor occurrence and development. This is of major importance for cell biological behavior regulation [33,34]. Current research has indicated that this signaling pathway is activated in human liver cancer tissues [35], lung cancer tissues [36], colorectal cancer tissues [37], ovarian cancer tissues [38], and other tumor tissues. At present, studies have shown that Ophiopogon saponins B pass through the Hippo pathway to induce cell apoptosis in NPC dependent on reactive oxygen, suggesting an involvement of the Hippo pathway NPC development [20]. In the research conducted this study, it was acquired that by interfering with lncRNA-LUADT1 expression, NPC cells saw an up-regulation in the LATS1 protein. The expression of YAP1 and TAZ was markedly down-regulated, inhibiting the activation of the Hippo/YAP pathway. However, low expression of miR-1207-5p can relieve this inhibitory effect. These results have revealed that lncRNA-LUADT1 can mediate Hippo/YAP pathway activity by regulating miR-1207-5p expression. However, the present study is still inadequate. Further studies are needed regarding the correlation between lncRNA-LUADT1 expression and clinicopathological characteristics of NPC patients, as well as the specific mechanism of lncRNA-LUADT1 promoting the NPC process *in vivo*.

## 5. Conclusion

To summarize, lncRNA-LUADT1 is highly expressed in NPC. lncRNA-LUADT1 competitively binds miR-1207-5p to regulate TEAD1, activates Hippo/YAP signaling pathway, and promotes proliferation, migration, and invasion of NPC cell lines, thereby aggravating NPC development, suggesting that lncRNA-LUADT1/miR-1207-5p/TEAD1 can be used as the target of NPC, providing innovative concepts for early disease diagnosis and treatment.

## Highlights

1. lncRNA-LUADT1 and TEAD1 expression are up-regulated in NPC, while miR-1207-5p is down-regulated.
2. lncRNA-LUADT1 low-expressed inhibits the proliferation, invasion, and migration of NPC cells.
3. TEAD1 is a target gene of miR-1207-5p.
4. 4) lncRNA-LUADT1 regulates miR-1207-5p/TEAD1 axis and inhibits the activation of Hippo/YAP signaling pathway.
